# Specific MiRNAs in naïve T cells associated with Hepatitis C Virus-induced Hepatocellular Carcinoma

**DOI:** 10.7150/jca.49594

**Published:** 2021-01-01

**Authors:** Peng Yan, Pengfei Pang, Xiaojun Hu, Ani Wang, Huitao Zhang, Yingdong Ma, Ke Zhang, Yaochao Ye, Bin Zhou, Junjie Mao

**Affiliations:** 1 Center for Interventional Medicine, the Fifth Affiliated Hospital, Sun Yat-sen University, Zhuhai, Guangdong 519000.; 2 Guangdong Provincial Key Laboratory of Biomedical Imaging, the Fifth Affiliated Hospital, Sun Yat-sen University, Zhuhai, Guangdong 519000.; 3 Guangdong Provincial Engineering Research Center of Molecular Imaging, the Fifth Affiliated Hospital, Sun Yat-sen University, Zhuhai, Guangdong 519000.; 4 Department of Cardiovascular Medicine, the Fifth Affiliated Hospital, Sun Yat-sen University, Zhuhai, Guangdong 519000, P.R. China.

**Keywords:** Hepatocellular carcinoma, hepatitis C virus, microRNAs, naïve T cells

## Abstract

Hepatocellular carcinoma (HCC) is the fifth most common type of cancer and the second leading cause of cancer-associated mortality worldwide. Hepatitis C virus (HCV) infection is the primary cause of hepatic fibrosis and cirrhosis, which in turn, notably increase the risk of developing HCC. The systematic immune response plays a vital role in protecting eukaryotic cells from exogenous antigens. In the present study, to determine the association between T cells and miRNAs in HCV-induced HCC (HCV-HCC), bulk mRNA and miRNA sequencing data from HCV-HCC tissues were combined, along with single-cell RNA sequencing (RNA-seq) data from T cells. Deconvoluted bulk RNA-seq data and miRNA profiles enabled the identification of naive CD4+ T cell-associated miRNAs, which may help to elucidate the underlying mechanism of the anti-HCV immune response. Using bulk RNA-seq data, the current analysis presents a feasible method for assessing the relationship between miRNAs and cell components, providing valuable insights into the effects of T cell-associated miRNAs in HCV-HCC.

## Introduction

Hepatocellular carcinoma (HCC) is the fifth most common cancer type, and the second leading cause of cancer-associated death worldwide 1. Hepatitis C virus (HCV) is a hepatotropic RNA virus, and infection with HCV is the principal cause of hepatic fibrosis and cirrhosis, the primary risk factors for HCC development 1. As the morbidity and mortality rates for HCV-induced cirrhosis and HCC remain high, elucidating the underlying mechanisms involved in HCV-induced HCC (HCV-HCC) is of great medical significance.

Immunotherapy has attracted increasing attention as an alternative approach to treating solid tumors. Immunotherapy enhances the recognition and cytotoxic immune response towards tumor cells 2. As critical components of the immune system, T cells exhibit helper, effector and memory functions 3. Since the liver is continuously exposed to microorganisms (including pathogens and the host gut flora), the hepatic environment establishes a balance between immunological tolerance (to maintain homeostasis) and an active immune response against invading pathogens 2.

The development of biotechnology has seen an increase in HCC transcriptomics research. As a result, bulk RNA sequencing (RNA-seq) has markedly improved our understanding of intratumor heterogeneity 4,5, clonal evolution 6 and metastatic dissemination 7,8 in tumors. Considering immunogenomic advancements 9 and the identification of biomarkers of prognosis and the therapeutic response 10, the use of RNA-seq has allowed a greater understanding of the tumor-immune microenvironment. Additionally, the use of single-cell sequencing (scRNA-seq) may help to deconstruct the complexities of the cellular immune microenvironment for the subsequent application of bulk RNA-seq.

MicroRNAs (miRNA/miRs) are small, non-coding single-stranded RNAs that post-transcriptionally modulate target gene expression, and participate in cellular processes, including proliferation, differentiation and apoptosis. Understanding the roles of miRNAs in monocyte heterogeneity provides insights into the association between miRNAs and specific cell types. As monocytes are involved in the pathogenesis of chronic inflammation, which is associated with the loss of tissue homeostasis and function, identifying monocyte-associated miRNAs may also aid the development of novel therapeutic strategies 11,12. Furthermore, miRNAs are also critically involved in immunoregulation 13. However, miRNA research is experimentally challenging at the single cell-level, which limits the use of this methodology. Identifying cell-specific miRNA subsets in diseased tissues is therefore an important step towards the discovery of potential diagnostic markers and therapeutic targets.

Numerous bioinformatics methods have recently been developed to analyze bulk RNA-seq data 14,15, including mRNAs and miRNAs; this may help to determine the potential function of miRNAs in specific cell components (CCs). In the present study, we hypothesized that gene expression was the same in each T cell component. Bulk RNA-seq measures the average expression of genes, which is the sum of different cell type-specific gene expression weighted by cell type proportions 14. Therefore, to identify CC-associated miRNAs, bulk RNA-seq data from HCV-HCC samples were deconvoluted using known T-cell single cell (sc)RNA-seq data as a reference, following by correlated with miRNA expression across the corresponding samples, providing valuable insights into the effects of T cell-associated miRNAs in HCV-HCC.

## Materials and methods

### Data collection

The scRNA-seq data of infiltrating T cells isolated from 6 patients with HCC were downloaded from the Gene Expression Omnibus (GEO) database 16 (dataset no. GSE98638 17). HCV-HCC-associated mRNA and miRNA RNA-seq data were also downloaded from the GEO (dataset no. GSE140845 and GSE140370, respectively), as well as the two validation independent datasets (dataset no. GSE82177 and GSE15421, respectively).

### Preprocessing raw mRNA and miRNA RNA-seq data

Adapters in raw sequencing files (.fq) were trimmed using *fastp* (v0.20.0) 18. The trimmed short reads from mRNAs and miRNAs were then aligned to the primary human genome assembly GRCh38 (hg38) from the Genome Reference Consortium using *HISAT2* (v2.1.0) 19 and *STAR* aligner (v2.7.1a) 20, respectively. Samples with low mapping rate (< 40%) were filtered out. *SAMtools* (v1.9) 21 was used to manipulate the mapped sequencing reads (.bam). *FeatureCounts* v2.0.0 (a software program of the *Subread* package) 22 was used to count reads to mRNAs and miRNAs in the Ensembl human gene annotation (v99) and miRBase (v22) 23 databases, respectively.

### Identification of cell type composition in HCV-HCC tissues

Using the scRNA-seq data of infiltrating T cells as a reference, the RNA-seq data of bulk HCV-HCC tissues were deconvoluted using the MUlti-Subject SIngle Cell deconvolution (MuSiC) method 14. Student's *t*-test was used to determine the significance of the difference between HCV-HCC and normal in each CC. *P*<0.05 was considered to indicate statistical significance.

### CCs of scRNA-seq data

The C01_CD8-LEF1 is dominant in the peripheral blood and specifically expressed naive marker genes such as LEF1 and CCR7. C03_CD8-SLC4A10, characterized by specific expression of SLC4A10, ZBTB16 and RORC, is largely composed of mucosal-associated invariant T cells, which are confirmed by the expression of semi-invariant TCR alpha chains with TRAV1-2/TRAJ33, TRAV1-2/TRAJ20 or TRAV1-2/TRAJ12. C04_CD8-LAYN, which is predominantly composed of cells from tumor tissues, expresses high levels of the exhaustion markers CTLA4, PDCD1 and HAVCR2, thus represents exhausted CD8+ T cells. For CD4+ T cells, C06_CD4-CCR7 comprise CD4+ cells with high expression of naive marker genes, including SELL, TCF7 and CCR7. C08_CD4-CTLA4 comprise FOXP3+ regulatory T cells, and expresses high levels of CTLA4. C09_CD4-GZMA expresses GZMA, and C10_CD4- CXCL13 specifically expresses CXCL13, PDCD1, CTLA4 and TIGIT, suggestive of an exhausted CD4+ T cell phenotype 17. Unclassified CC was defined as cells without T cell-related markers, which are regarded as “other cell types”.

### Differential expression analysis

DEGs between the HCV-HCC and normal groups were detected using an R/Bioconductor package *DESeq2* (v1.26.0) 24. Multiple testing correction was performed using the Benjamini-Hochberg method and false discovery rate (FDR). Genes with an FDR<0.1 were identified as significantly differentially expressed.

### Correlation analysis and plotting

Co-expression analysis between miRNAs and CCs was performed using Pearson's Correlation analysis in the Python package *scipy* (v1.4.1). *P*<0.05 was considered to indicate a significant correlation between miRNAs and CCs. Plots were generated using the Python package *seaborn* (v0.10.0) and the R/Bioconductor *ggplot* (v3.1.1) and *complexheatmap* (v2.4.2) 25 packages.

### Chromosomal location, disease, Gene Ontology (GO) term, and pathway enrichment analysis

miRNA functional enrichment analysis was performed using miEAA (v2.0) 26, which detects significantly enriched chromosomal locations, diseases, and GO terms based on gene annotations from miRbase (v22) 23, MNDR 27, and GO 28. Adjusted *P*-values using Benjamini-Hochberg method that <0.05 was considered to indicate statistical significance. Gene set enrichment analysis (GSEA) 29,30 was performed to detect significantly enriched Kyoto Encyclopedia of Genes and Genomes (KEGG) 31 pathways.

### Target predictions of miRNAs

Targets of miRNAs were predicted by algorithms including PITA 32, RNA22 33, miRmap 34, microT 35, miRanda 36, PicTar 37, and TargetScan 38, which have been implemented in starBase v3.0 39. Targets that predicted by not less than 4 of these algorithms were considered as high-confidence. KEGG pathways and GO term enrichment analysis of the predicted targets were performed by the R/Bioconductor *clusterprofiler* (v3.16.0) package 40. Adjusted *P*-values using Benjamini-Hochberg method that <0.05 was considered to indicate statistical significance.

## Results

### Increased proportions of naïve T cells in HCV-HCC

**Figure 1:**
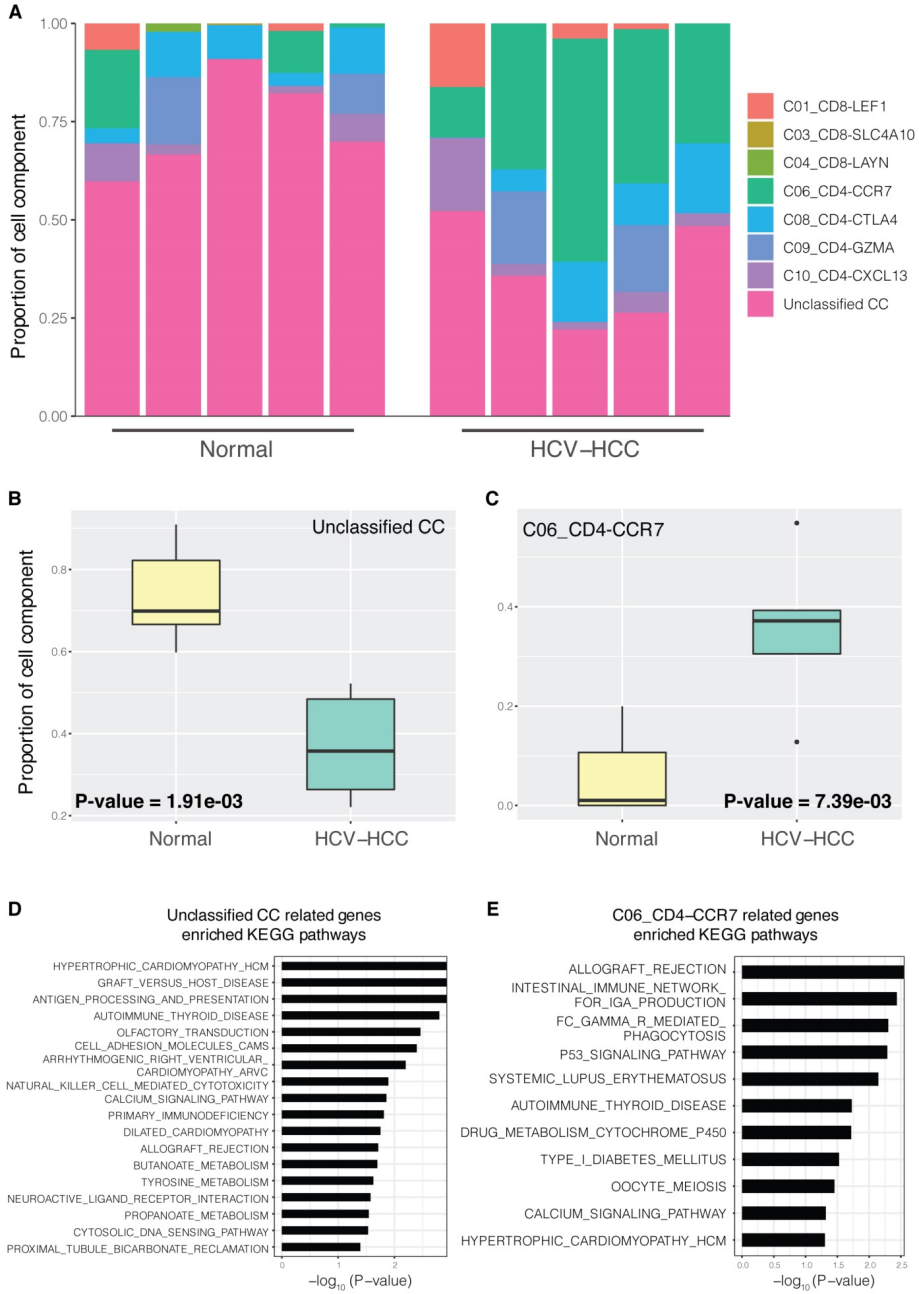
** CCs in HCV-HCC and normal control tissues.** (**A**) Proportion of CCs in different samples; normal control group (left) and HCV-HCC group (right); n=5 per group. Each bar represents one sample, and different colors in each bar represent a single cell component. (**B and C**) Boxplot of significantly different CC proportions between the HCV-HCC and normal control groups (two-tailed t-test; *P*<0.05). (**B**) Unclassified CC. (C) C06_CD4-CCR7 cell component. (**D and E**) KEGG pathway enrichment results for two significantly different CCs; the x-axis represents -log10 (*P*-value), where P is the significant value for enrichment. KEGG pathway names are presented on the y-axis. (**D**) Unclassified CC. (**E**) C06_CD4-CCR7. CC, cell component; HCV-HCC, HCV-induced HCC; KEGG, Kyoto Encyclopedia of Genes and Genomes.

Generally, only differentially expressed genes between groups, but not CCs of each tissue sample, could be detected using bulk RNA-seq data. Using scRNA-seq data and MuSiC (detailed in the Materials and methods), the bulk HCV-HCC RNA-seq data were decomposed into CCs using various T cell signatures. CCs varied significantly between the different sample groups (Fig. 1A). Notably, C06_CD4-CCR7 and unclassified CCs showed significant difference between the HCV-HCC and normal groups (Fig. 1B; *P*=1.91×10^-3^ and *P*=7.39×10^-3^, respectively). CCs without significant differences are displayed in Fig. S1. We then investigated the relation between the proliferation marker *MKI67* and CCs. The Pearson's correlation coefficient between *MKI67* and CCs (Table SI) showed the only significant positive correlation in the gene expression of *MKI67* and CD4-CCR7 naïve T cells (*P*=2.37×10^-2^). Moreover, gene expression of *MKI67* is upregulated in HCV-HCC liver tissue (Fig. S2). Therefore, the higher proportion of C06_CD4-CCR7 between the HCV-HCC and normal groups represented a potentially more proliferative naïve T cell population in the HCV-HCC group. In “other diseases” (such as leishmaniasis), a higher count of CCR7+ naïve T cells was also reported, compared with those of the normal groups 41, indicating greater proliferative potential in these diseases. GSEA enrichment analysis (detailed in the Materials and methods) for the C06_CD4-CCR7 revealed a close relationship between pathways of the immune system and cancer (Fig. 1E). For example, consistent with the known finding that HCV infection may cause the acute rejection of transplanted kidneys 42, the increased proportion of C06_CD4-CCR7 in HCV-HCC was also shown to be involved in allograft rejection.

### Limited numbers of overlapping miRNAs were detected between CCs and differentially expressed (DE) miRNAs

A total of 200 miRNAs (Table SII) were found to correlate with 8 CCs (unclassified, C01_CD8-LEF1, C03_CD8-SLC4A10, C04_CD8-LAYN, C06_CD4-CCR7, C09_CD4-GZMA, C08_CD4-CTLA4 and C10_CD4-CXCL13). The number of CC-related miRNAs are listed in Table 1, with the number of positive correlations listed in brackets. miRNAs that only correlated with one CC were defined as unique miRNAs. For example, C09_CD4-GZMA had the highest number of unique miRNAs, and 41 out of 43 were positively correlated, indicating that these miRNAs may exclusively function in C09_CD4-GZMA.

**Figure 2:**
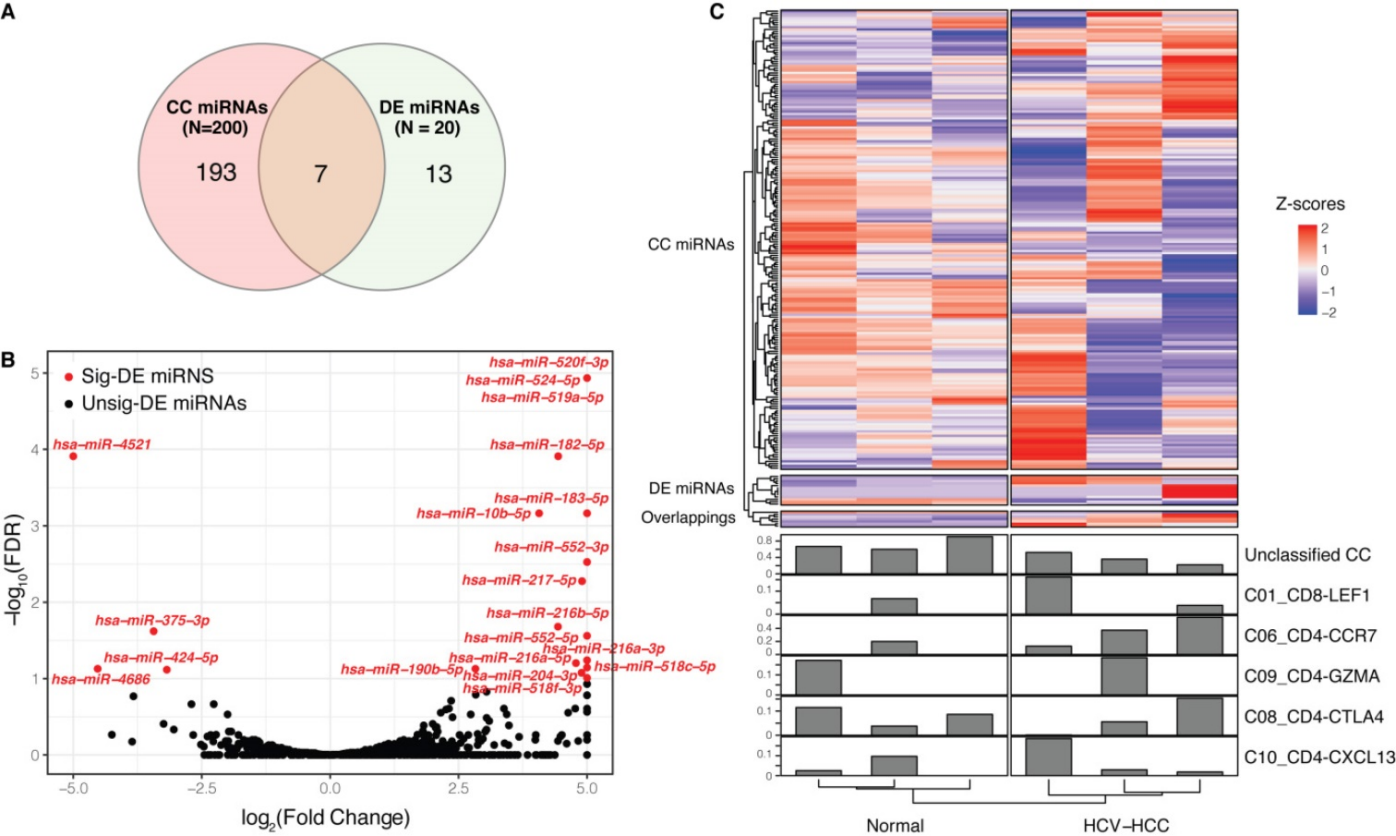
** Correlation between miRNAs and CCs.** (**A**) Venn diagram of DE miRNAs and CC-related miRNAs. (**B**) Volcano plot of DE miRNAs between the HCV-HCC and normal control groups. Significantly DE miRNAs (FDR<0.1) are red dots with miRNA names, while black dots represent those that are not DE; the x-axis represents the log_2_(fold change) and the y axis is the -log_10_(FDR). (**C**) Complex heatmap of miRNA expression and CCs across samples. A total of 213 miRNAs are represented in the upper heatmap, whose type was determined according to (A). Each line in the bottom bar plots represents the proportion of each CC across the samples. CC, cell component; miRNA, microRNA; DE, differentially expressed; FDR, false discovery rate.

Furthermore, 20 DE miRNAs were identified between the HCV-HCC and normal groups, which showed limited overlap with CC miRNAs (Fig. 2A-C, Table SIII); these included hsa-miR-10b-5p, -183-5p, -190b-5p, -204-3p, -216a-5p, -4521 and -552-5p. Among these CC-DE miRNAs, hsa-miR-190b-5p was correlated with two CCs, namely C06_CD4-CCR7 and unclassified. hsa-miR-190b-5p has been proven to serve as an initiation and progression factor in breast cancer 43. In the present study, hsa-miR-190b-5p was consistently expressed in combination with C06_CD4-CCR7 across all samples, indicating a potential role for naïve T cell-associated hsa-miR-190b-5p in the development of HCV-HCC. Moreover, three other overlapping miRNAs (hsa-miR-10b-5p, hsa-miR-204-3p and hsa-miR-552-5p) were also correlated with C06_CD4-CCR7, which has been associated with estrogen receptor-positive breast cancer 44 and colon cancer 45-47. hsa-miR-4521, hsa-miR-183-5p and hsa-miR-216a- 5p, which were identified as significantly DE miRNAs, also act as oncogenes in renal cell carcinoma 48-50 and bladder cancer 51. These miRNAs were not difficult to detect, as their expression levels varied considerably between cancerous and normal tissues.

### Key roles of C06_CD4-CCR7 T cell-associated miRNAs in multiple cancer types

**Figure 3:**
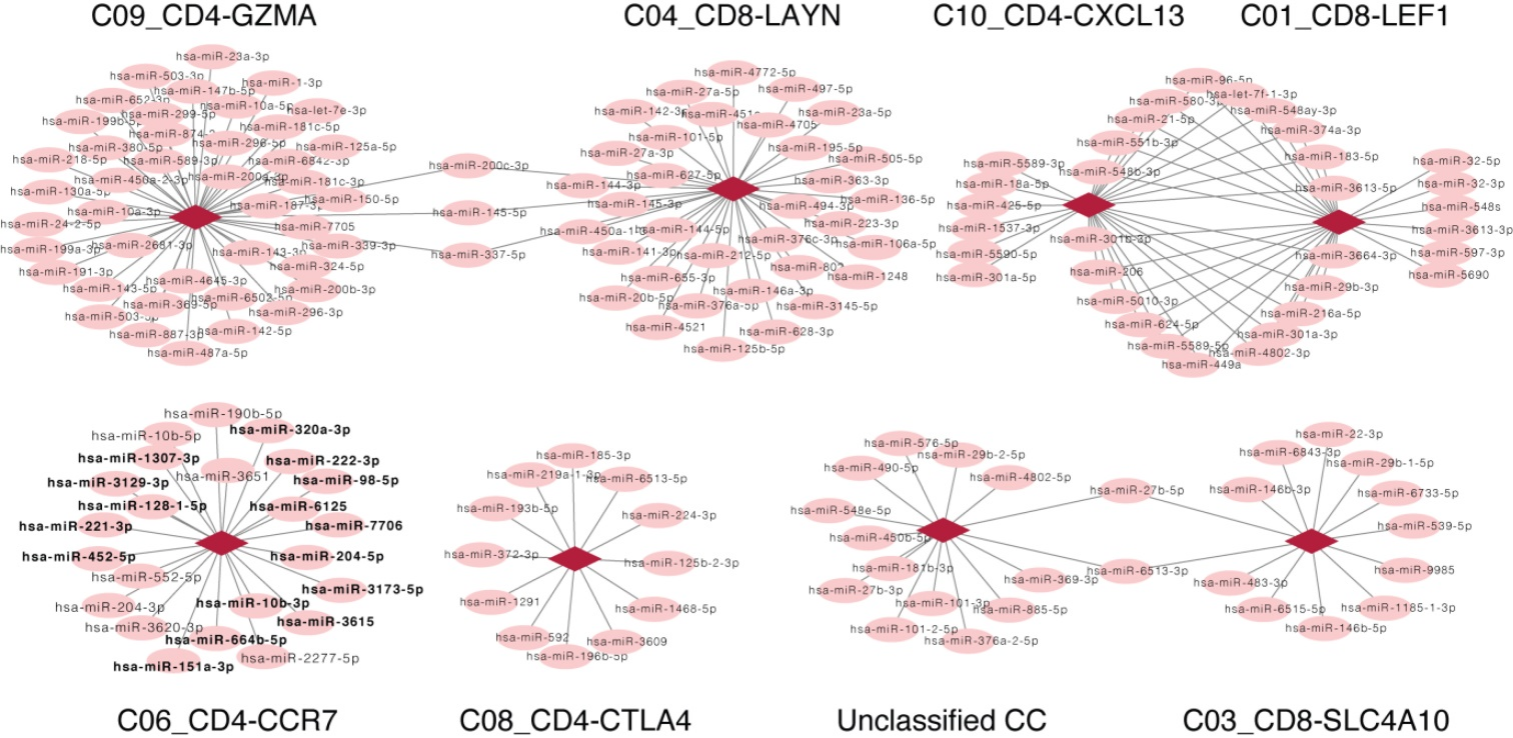
** Network representing related CCs and miRNAs.** Each circle represents a miRNA, and each diamond represents a different CC. Lines between nodes represent CCs and miRNAs which consistently differed across samples (Pearson's correlation; *P*<0.05). There is no line if the change of the CC and miRNA displayed opposing the trends. Except for DE miRNAs, unique miRNAs that positively correlated with C06_CD4-CCR7 were highlighted in bold. CC, cell component; miRNA, microRNA.

As the fixed proportion of genes in one CC, a positive relation, (indicating that the increased number of miRNAs is consistent with the increased proportion of CCs), suggests that the miRNAs were involved in the CC. On the contrary, a negative relation suggests a non-inclusive association between miRNAs and CCs, which would not be further analyzed. Here, we focused only on miRNAs that were positively related to CCs; a correlation network was subsequently constructed based on the positive correlation between CCs and miRNA expression, including 199 positive correlations between 173 miRNAs and 8 CCs (Fig. 3). Besides DE miRNAs, another considerable miRNA population was found to exclusively correlate with CCs that may serve vital roles in HCV-HCC. Enrichment analysis was performed for such miRNAs that associated with C06_CD4-CCR7 (highlighted in Fig. 3).

Among the miRNAs that correlated with C06_CD4-CCR7, hsa-miR-452-5p, hsa-miR-222-3p, hsa-miR-664b-5p, hsa-miR-221-3p, and hsa-miR-98-5p were enriched in chromosome X (FDR=2.62×10^-2^, Table SIV). As chromosome X contains a high density of immune-related genes and regulatory elements that are extensively involved in both the innate and adaptive immune responses 52, we could infer that these miRNAs play vital roles in directly function or indirectly regulate immune system.

Although these C06_CD4-CCR7-associated miRNAs did not show significantly differential expression in HCV-HCC, they were detected as significantly enriched in different cancer types, implying an underlying relationship between these miRNAs and cancers (Table SIV). Thus, C06_CD4-CCR7, the naïve T cells might play key role in microenvironments of various types of cancers. For example, as reported in breast cancer, blocking the recruitment of naïve CD4+ T cells into tumor significantly reduces intratumoral regulatory T cells and inhibits tumor progression 53.

Furthermore, these C06_CD4-CCR7-associated miRNAs were significantly enriched in negative regulation by host of viral genome replication (FDR= 4.32×10^-3^), indicating only few naïve T cells would differentiate to maturity and interfere with virus proliferation or immigration.

In summary, the aforementioned miRNAs serve key roles in different types of cancer, and their upregulation was consistent with the C06_CD4-CCR7. The data indicate that these miRNAs are involved in C06_CD4-CCR7, and serve important roles in C06_CD4-CCR7 in patients with HCV-HCC.

### Validation of the increasing trend of C06_CD4-CCR7 in two independent HCV-HCC datasets

The same analysis pipeline was performed to two independent HCC datasets. In GSE82177 dataset, the mean proportion of C06_CD4-CCR7 was 50.18% in 9 normal samples as compared to 55.51% in HCV-HCC samples (Fig. S3A), indicating an increasing proportion of C06_CD4-CCR7 in HCV-HCC than normal ones. In GSE154211 dataset, the proportion of C06_CD4-CCR7 was higher in HCV-HCC samples comparing to the paired non-tumor samples (Fig. S3B). Taken together, be in line with our findings, the validation datasets showed an increased proportion of C06_CD4-CCR7, the naïve T cells in HCV-HCC.

## Discussion

Immunotherapy is one of the current methods for the treatment of cancer. For those suffering with cancer, immunotherapeutic developments have accelerated the translation of immunological knowledge into medical breakthroughs. However, research has predominantly focused on transcriptomics, with few studies considering immunotherapy beyond regulation of the transcriptome.

The present study outlines a pilot analysis that provides a glimpse into the correlation between miRNAs and specific T cell-associated CCs. The expression of various miRNAs has been reported to correlate with tumor progression and proliferation, but few studies have considered the associated cell types. CD4+ T cells represent a unique branch of the adaptive immune system that is crucial for the effective regulation of antipathogenic responses, thus their function is vital for survival. With distinct phenotypes according to their respective cytokine profiles, CD4+ T cells modulate the functions of innate immune cells as well as members of the adaptive immune system. Thus, elucidating the relationship between miRNAs and T cells may facilitate the therapeutic regulation of the immune system.

In the present study, CD4+ naïve T cells were the principal CC differing between HCV-HCC and normal liver tissues. This indicates that during HCV infection, naïve T cells would proliferate, but few would differentiate to maturity and interfere with virus proliferation or immigration. T cells must be activated in order to differentiate, which is a topic for further research. Other CCs, except for the unclassified CC, showed no significant difference between HCV-HCC and normal liver tissues. For example, because of the proliferation but no differentiation of the CD4+ naïve T cells, the CD4+ regulatory T cells (C08_CD4-CTLA4), which are thought to be derived from the same lineage as the CD4+ naïve T cells 54, showed the similar proportion in HCV-HCC and normal liver tissue.

In our study, we only focused on the C06_CD4-CCR7-associated miRNAs, which serve key roles in different types of cancer, while other CC-associated miRNAs would not be discussed in this work. For example, hsa-miR-7706 was revealed to be a prognostic marker in HCC. Downregulation of hsa-miR-7706 was found to inhibit the proliferation of HCC cells, and may potentially be used as a novel target for the treatment of HCC 55. In the present study, hsa-mir-7706 expression was upregulated in the HCV-HCC group, and was consistently present with C06_CD4-CCR7, indicating the potential activation of C06_CD4-CCR7 in HCC. Although no previous studies have reported the relationship between hsa-miR-10b-3p and HCC, this miRNA has been found to be significantly upregulated in the tumor tissues and serum samples of patients with esophageal squamous cell carcinoma 56. Due to the correlation between hsa-miR-10b-3p and C06_CD4-CCR7 identified in the present study, hsa-miR-10b-3p could be inferred as a biomarker of naive T cells, where it may induce the progression of cancer in general. Moreover, hsa-miR-10b-3p has also been identified as a prognostic biomarker of overall survival in colorectal cancer 57.

Another correlated miRNA (hsa-miR-151a-3p) has been found to inhibit LPS-induced interleukin (IL)-6 production by targeting Stat3 58. As IL-6 is critical for the signal transduction and subsequent function of cytokines, as well as the production of pro-inflammatory cytokines, hsa-miR-151a-3p may influence the regulation of innate immunity and inflammation. hsa-miR-98-5p has also been revealed to inhibit proliferation and metastasis in non-small cell lung cancer 59. In chemotherapy-resistant epithelial ovarian cancer, hsa-miR-1307-3p was significantly differentially expressed 60. hsa-miR-221-3p and hsa-miR-222-3p were found to be widely distributed in eukaryotic organisms and critically involved in posttranscriptional gene regulation. Their expression levels are also closely associated with tumor stage and prognosis. Thus, the combined expression of these two miRNAs has been suggested as a biomarker for the diagnosis of premalignant tumors, as well as a novel target for tumor therapy, and a therapeutic tool for drug resistance or sensitivity to anticancer treatment 61. Elevated hsa-miR-222-3p expression may promote the proliferation and invasion of endometrial carcinoma by targeting estrogen receptor (ER) 62. In Kawasaki disease, platelet-associated hsa-miR-222-3p serves as a distinguishing marker for early recognition, based on its significant upregulation in the platelets of patients in the acute stages of disease. Furthermore, Kyoto Encyclopedia of Genes and Genomes pathway analysis revealed that targets of miR-222-3p are enriched in immune-related signaling pathways 63.

hsa-miR-320a-3p serves as a negative regulator in the progression of gastric cancer by targeting RAB14. The reintroduction of RAB14 partially abrogated its miR-320a-mediated downregulation and reversed the miR-320a-induced effects on gastric cancer cell proliferation 64.

The targets of hsa-miR-222-3p and hsa-miR-221-3p, two C06_CD4-CCR7 related miRNAs, were significantly enriched in the cellular senescence pathway (KEGG: hsa04218, FDR=4.03×10^-5^ for hsa-miR-222-3p, FDR=7.75×10^-4^ for hsa-miR-221-3p). Factors that drive T cell differentiation and senescence were related 65. Cellular senescence might be one of the factors in the inhibition of naïve T cell differentiation. Besides, targets of hsa-miR-222-3p were highly associated with mitochondrial functions, protein insertion into mitochondrial membrane (biology process: GO:0051204, FDR=2.65×10^-5^), establishment of protein localization to mitochondrial membrane (biology process: GO:0090151, FDR=2.99×10^-5^), positive regulation of mitochondrial membrane permeability (biology process: GO:0035794, FDR=9.32×10^-5^). Mitochondrial mass was proved as increasing for rapid proliferation during differentiation 66,67, indicating that hsa-miR-222-3p played potential role in naïve T cell proliferation and differentiation.

The results of the present study were generated with bioinformatics methods, thus further experimental validation is required to support these conclusions. As a biotechnological limitation, high-throughput miRNA profile scanning in single cells is difficult to conduct. The current strategy provides a means of analyzing bulk RNA-seq and corresponding small RNA-seq data, which may reveal novel conclusions from previous datasets, and the correlation between miRNAs and corresponding CCs.

Based on high-throughput scanning and low-throughput validation, increasing research into the correlation between miRNAs and specific types of cancer cells may help to clarify the effects of post-transcriptome regulation in heterogeneity, evolution and drug resistance.

## Supplementary Material

Supplementary figures and tables.

## Figures and Tables

**Table 1:** The number of cell components-related miRNAs

Cell components	CC-related miRNAs (positive)	Unique miRNAs (positive)
C01_CD8-LEF1	36 (27)	14 (6)
C03_CD8-SLC4A10	14 (13)	10 (10)
C04_CD8-LAYN	37 (37)	34 (34)
C06_CD4-CCR7	45 (23)	33 (18)
C08_CD4-CTLA4	14 (12)	12 (12)
C09_CD4-GZMA	47 (45)	43 (41)
C10_CD4-CXCL13	28 (27)	6 (6)
Unclassified CC	22 (15)	7 (6)

## Abbreviations

CC: cell component
DE: differentially expressed
DEGs: differentially expressed genes
FDR: false discovery rate
GEO: Gene Expression Omnibus
HCC: hepatocellular carcinoma
HCV: Hepatitis C virus
HCV-HCC: HCV-induced HCC
KEGG: Kyoto Encyclopedia of Genes and Genomes
miRNA: microRNA
RNA-seq: RNA sequencing
scRNA-seq: single-cell RNA sequencing

## References

[1] Axley P, Ahmed Z, Ravi S, Singal AK (2018). Hepatitis C Virus and Hepatocellular Carcinoma: A Narrative Review. J Clin Transl Hepatol.

[2] Aravalli R, Steer C (2017). Immune-Mediated Therapies for Liver Cancer. Genes (Basel).

[3] Kumar B V, Connors TJ, Farber DL (2018). Human T Cell Development, Localization, and Function throughout Life. Immunity.

[4] Liu Y, Yang Z, Du F, Yang Q, Hou J, Yan X (2017). Molecular mechanisms of pathogenesis in hepatocellular carcinoma revealed by RNA-sequencing. Mol Med Rep.

[5] Jin Y, Lee WY, Toh ST, Tennakoon C, Toh HC, Chow PK-H (2019). Comprehensive analysis of transcriptome profiles in hepatocellular carcinoma. J Transl Med.

[6] Zhou Z, Xu B, Minn A, Zhang NR (2019). Genetic Heterogeneity Profiling by Single Cell RNA Sequencing. bioRxiv.

[7] Wingrove E, Liu ZZ, Patel KD, Arnal-Estapé A, Cai WL, Melnick M-A (2019). Transcriptomic Hallmarks of Tumor Plasticity and Stromal Interactions in Brain Metastasis. Cell Rep.

[8] Karaayvaz M, Cristea S, Gillespie SM, Patel AP, Mylvaganam R, Luo CC (2018). Unravelling subclonal heterogeneity and aggressive disease states in TNBC through single-cell RNA-seq. Nat Commun.

[9] Saito R, Smith CC, Utsumi T, Bixby LM, Kardos J, Wobker SE (2018). Molecular Subtype-Specific Immunocompetent Models of High-Grade Urothelial Carcinoma Reveal Differential Neoantigen Expression and Response to Immunotherapy. Cancer Res.

[10] Hellmann MD, Callahan MK, Awad MM, Calvo E, Ascierto PA, Atmaca A (2018). Tumor Mutational Burden and Efficacy of Nivolumab Monotherapy and in Combination with Ipilimumab in Small-Cell Lung Cancer. Cancer Cell.

[11] Duroux-Richard I, Robin M, Peillex C, Apparailly F (2019). MicroRNAs: Fine Tuners of Monocyte Heterogeneity. Front Immunol. 2019;10. doi:10.3389/fimmu.

[12] Lindsay MA (2008). microRNAs and the immune response. Trends Immunol.

[13] Raisch J (2013). Role of microRNAs in the immune system, inflammation and cancer. World J Gastroenterol.

[14] Wang X, Park J, Susztak K, Zhang NR, Li M (2019). Bulk tissue cell type deconvolution with multi-subject single-cell expression reference. Nat Commun.

[15] Newman AM, Steen CB, Liu CL, Gentles AJ, Chaudhuri AA, Scherer F (2019). Determining cell type abundance and expression from bulk tissues with digital cytometry. Nat Biotechnol.

[16] Edgar R (2002). Gene Expression Omnibus: NCBI gene expression and hybridization array data repository. Nucleic Acids Res.

[17] Zheng C, Zheng L, Yoo J-K, Guo H, Zhang Y, Guo X (2017). Landscape of Infiltrating T Cells in Liver Cancer Revealed by Single-Cell Sequencing. Cell.

[18] Chen S, Zhou Y, Chen Y, Gu J (2018). fastp: an ultra-fast all-in-one FASTQ preprocessor. Bioinformatics.

[19] Kim D, Paggi JM, Park C, Bennett C, Salzberg SL (2019). Graph-based genome alignment and genotyping with HISAT2 and HISAT-genotype. Nat Biotechnol.

[20] Dobin A, Davis CA, Schlesinger F, Drenkow J, Zaleski C, Jha S (2013). STAR: ultrafast universal RNA-seq aligner. Bioinformatics.

[21] Li H, Handsaker B, Wysoker A, Fennell T, Ruan J, Homer N (2009). The Sequence Alignment/Map format and SAMtools. Bioinformatics.

[22] Liao Y, Smyth GK, Shi W (2013). The Subread aligner: fast, accurate and scalable read mapping by seed-and-vote. Nucleic Acids Res.

[23] Kozomara A, Birgaoanu M, Griffiths-Jones S (2019). miRBase: from microRNA sequences to function. Nucleic Acids Res.

[24] Love MI, Huber W, Anders S (2014). Moderated estimation of fold change and dispersion for RNA-seq data with DESeq2. Genome Biol.

[25] Gu Z, Eils R, Schlesner M (2016). Complex heatmaps reveal patterns and correlations in multidimensional genomic data. Bioinformatics.

[26] Kern F, Fehlmann T, Solomon J, Schwed L, Grammes N, Backes C (2020). miEAA 2.0: integrating multi-species microRNA enrichment analysis and workflow management systems. Nucleic Acids Res.

[27] Cui T, Zhang L, Huang Y, Yi Y, Tan P, Zhao Y (2017). MNDR v2.0: an updated resource of ncRNA-disease associations in mammals. Nucleic Acids Res.

[28] Ashburner M, Ball CA, Blake JA, Botstein D, Butler H, Cherry JM (2000). Gene Ontology: tool for the unification of biology. Nat Genet.

[29] Subramanian A, Tamayo P, Mootha VK, Mukherjee S, Ebert BL, Gillette MA (2005). Gene set enrichment analysis: A knowledge-based approach for interpreting genome-wide expression profiles. Proc Natl Acad Sci.

[30] Mootha VK, Lindgren CM, Eriksson K-F, Subramanian A, Sihag S, Lehar J (2003). PGC-1α-responsive genes involved in oxidative phosphorylation are coordinately downregulated in human diabetes. Nat Genet.

[31] Kanehisa M (2000). KEGG: Kyoto Encyclopedia of Genes and Genomes. Nucleic Acids Res.

[32] Kertesz M, Iovino N, Unnerstall U, Gaul U, Segal E (2007). The role of site accessibility in microRNA target recognition. Nat Genet.

[33] Fahlgren N, Carrington JC (2010). miRNA Target Prediction in Plants. Methods Mol Biol.

[34] Vejnar CE, Zdobnov EM (2012). MiRmap: comprehensive prediction of microRNA target repression strength. Nucleic Acids Res.

[35] Paraskevopoulou MD, Georgakilas G, Kostoulas N, Vlachos IS, Vergoulis T, Reczko M (2013). DIANA-microT web server v5.0: service integration into miRNA functional analysis workflows. Nucleic Acids Res.

[36] John B, Enright AJ, Aravin A, Tuschl T, Sander C, Marks DS (2004). Human MicroRNA targets. PLoS Biol.

[37] Krek A, Grün D, Poy MN, Wolf R, Rosenberg L, Epstein EJ (2005). Combinatorial microRNA target predictions. Nat Genet.

[38] Lewis BP, Burge CB, Bartel DP (2005). Conserved seed pairing, often flanked by adenosines, indicates that thousands of human genes are microRNA targets. Cell.

[39] Li J-H, Liu S, Zhou H, Qu L-H, Yang J-H (2014). starBase v2.0: decoding miRNA-ceRNA, miRNA-ncRNA and protein-RNA interaction networks from large-scale CLIP-Seq data. Nucleic Acids Res.

[40] Yu G, Wang L-G, Han Y, He Q-Y (2012). clusterProfiler: an R package for comparing biological themes among gene clusters. OMICS.

[41] Keshavarz Valian H, Nateghi Rostami M, Tasbihi M, Miramin Mohammadi A, Eskandari SE, Sarrafnejad A (2013). CCR7+ Central and CCR7- Effector Memory CD4+ T Cells in Human Cutaneous Leishmaniasis. J Clin Immunol.

[42] Karkout K, Al Sherif S, Hussein Q, Albawardi A, Boobes Y (2018). Possible acute rejection associated with the use of the new anti-hepatitis C virus medications. Avicenna J Med.

[43] Dai W, He J, Zheng L, Bi M, Hu F, Chen M (2019). miR-148b-3p, miR-190b, and miR-429 Regulate Cell Progression and Act as Potential Biomarkers for Breast Cancer. J Breast Cancer.

[44] Cizeron-Clairac G, Lallemand F, Vacher S, Lidereau R, Bieche I, Callens C (2015). MiR-190b, the highest up-regulated miRNA in ERα-positive compared to ERα-negative breast tumors, a new biomarker in breast cancers?. BMC Cancer.

[45] Xi X, Teng M, Zhang L, Xia L, Chen J, Cui Z (2020). MicroRNA-204-3p represses colon cancer cells proliferation, migration, and invasion by targeting HMGA2. J Cell Physiol.

[46] Jin H, Wang X, Chen L, Zhang Y, Tang X, Tang G (2016). Screening miRNAs for early diagnosis of colorectal cancer by small RNA deep sequencing and evaluation in a Chinese patient population. Onco Targets Ther.

[47] Wang J, Li H, Wang Y, Wang L, Yan X, Zhang D (2016). MicroRNA-552 enhances metastatic capacity of colorectal cancer cells by targeting a disintegrin and metalloprotease 28. Oncotarget.

[48] Zhang X, Xu G, Zhou Y, Yan J (2018). MicroRNA-183 promotes the proliferation and metastasis of renal cell carcinoma through targeting Dickkopf-related protein 3. Oncol Lett.

[49] Chen P, Quan J, Jin L, Lin C, Xu W, Xu J (2018). miR-216a-5p acts as an oncogene in renal cell carcinoma. Exp Ther Med.

[50] Feng X, Yan N, Sun W, Zheng S, Jiang S, Wang J (2019). miR-4521-FAM129A axial regulation on ccRCC progression through TIMP-1/MMP2/MMP9 and MDM2/p53/Bcl2/Bax pathways. Cell Death Discov.

[51] Gao J-M, Huang L-Z, Huang Z-G, He R-Q (2018). Clinical value and potential pathways of miR-183-5p in bladder cancer: A study based on miRNA-seq data and bioinformatics analysis. Oncol Lett.

[52] Schurz H, Salie M, Tromp G, Hoal EG, Kinnear CJ, Möller M (2019). The X chromosome and sex-specific effects in infectious disease susceptibility. Hum Genomics.

[53] Thol F, Klesse S, Köhler L, Gabdoulline R, Kloos A, Liebich A (2017). Acute myeloid leukemia derived from lympho-myeloid clonal hematopoiesis. Leukemia.

[54] Curiel TJ (2007). Tregs and rethinking cancer immunotherapy. J Clin Invest.

[55] Wang F, Dai M, Chen H, Li Y, Zhang J, Zou Z (2018). Prognostic value of hsa-mir-299 and hsa-mir-7706 in hepatocellular carcinoma. Oncol Lett.

[56] Lu Y, Yu J, Yang Z, Zhu G, Gao P, Wang H (2018). Promoter hypomethylation mediated upregulation of MicroRNA-10b-3p targets FOXO3 to promote the progression of esophageal squamous cell carcinoma (ESCC). J Exp Clin Cancer Res.

[57] Yang G, Zhang Y, Yang J (2019). A Five-microRNA Signature as Prognostic Biomarker in Colorectal Cancer by Bioinformatics Analysis. Front Oncol. 2019;9. doi:10.3389/fonc.

[58] Liu X, Su X, Xu S, Wang H, Han D, Li J (2018). MicroRNA *in vivo* precipitation identifies miR-151-3p as a computational unpredictable miRNA to target Stat3 and inhibits innate IL-6 production. Cell Mol Immunol.

[59] Jiang F, Yu Q, Chu Y, Zhu X, Lu W, Liu Q (2018). MicroRNA-98-5p inhibits proliferation and metastasis in non-small cell lung cancer by targeting TGFBR1. Int J Oncol.

[60] Zhou Y, Wang M, Wu J, Jie Z, Chang S, Shuang T (2015). The clinicopathological significance of miR-1307 in chemotherapy resistant epithelial ovarian cancer. J Ovarian Res.

[61] Song Q, An Q, Niu B, Lu X, Zhang N, Cao X (2019). Role of miR-221/222 in Tumor Development and the Underlying Mechanism. J Oncol.

[62] Liu B, Che Q, Qiu H, Bao W, Chen X, Lu W (2014). Elevated MiR-222-3p Promotes Proliferation and Invasion of Endometrial Carcinoma via Targeting ERα. PLoS One.

[63] Wang B, Wang L, Cheng F, Lv H, Sun L, Wei D (2019). MiR-222-3p in Platelets Serves as a Distinguishing Marker for Early Recognition of Kawasaki Disease. Front Pediatr. 2019;7. doi:10.3389/fped.

[64] Li Y, Liu H, Shao J, Xing G (2017). miR-320a serves as a negative regulator in the progression of gastric cancer by targeting RAB14. Mol Med Rep.

[65] Goronzy JJ, Weyand CM (2019). Mechanisms underlying T cell ageing. Nat Rev Immunol.

[66] Tyrakis PA, Palazon A, Macias D, Lee KL, Phan AT, Veliça P (2016). S-2-hydroxyglutarate regulates CD8+ T-lymphocyte fate. Nature.

[67] van der Windt GJW, Everts B, Chang C-H, Curtis JD, Freitas TC, Amiel E (2012). Mitochondrial respiratory capacity is a critical regulator of CD8+ T cell memory development. Immunity.

