# Supplementary figures and images for: Specific MiRNAs in naïve T cells associated with Hepatitis C Virus-induced Hepatocellular Carcinoma
Source: J Cancer. 2021 Jan 1;12(1):1–9. doi: 10.7150/jca.49594 (PMC7738825; doi:10.7150/jca.49594)

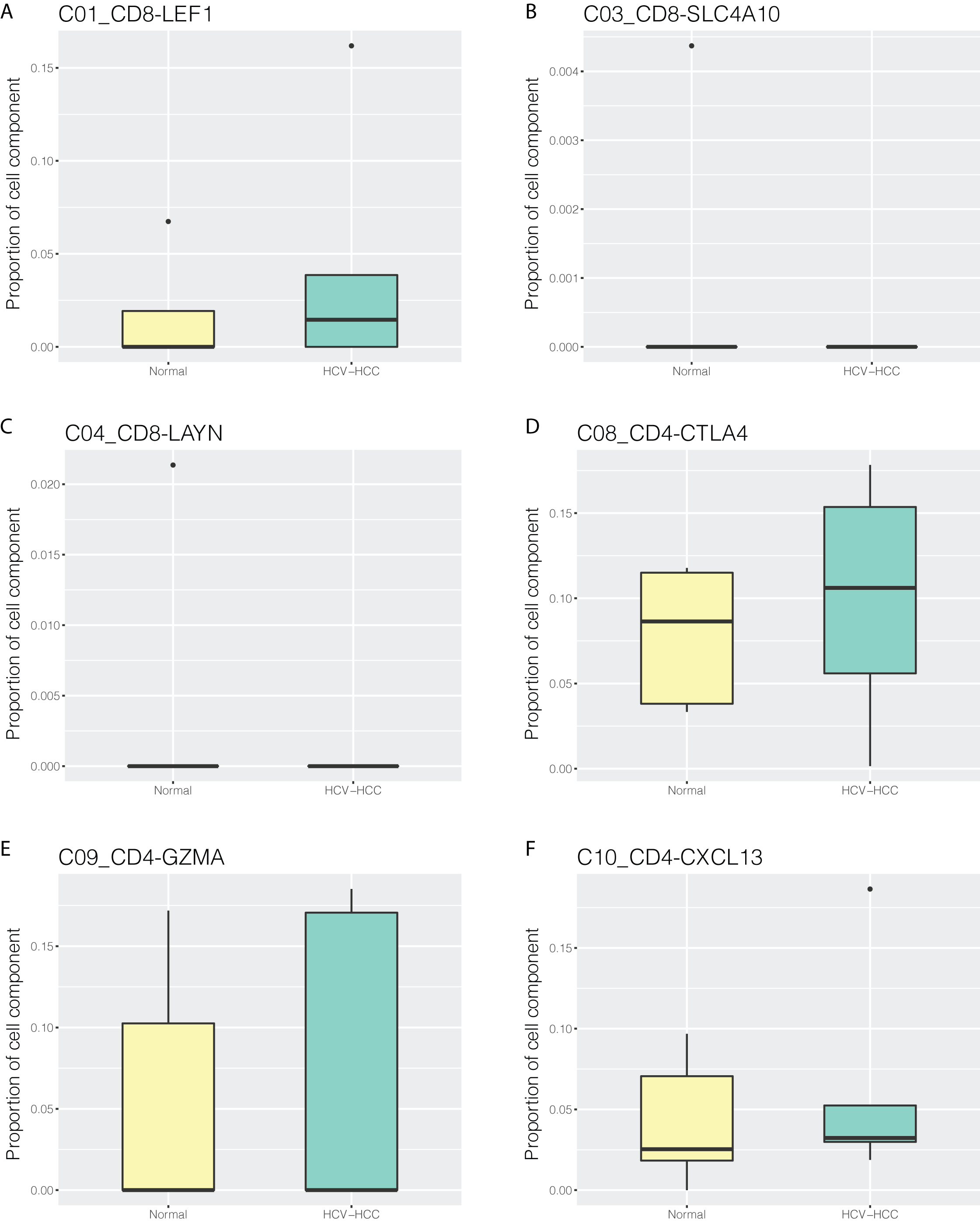

Supplement: Supplementary file 1 — Supplementary figures and tables. [file jcav12p0001s1.zip › Supplementary Figure S1.tif]

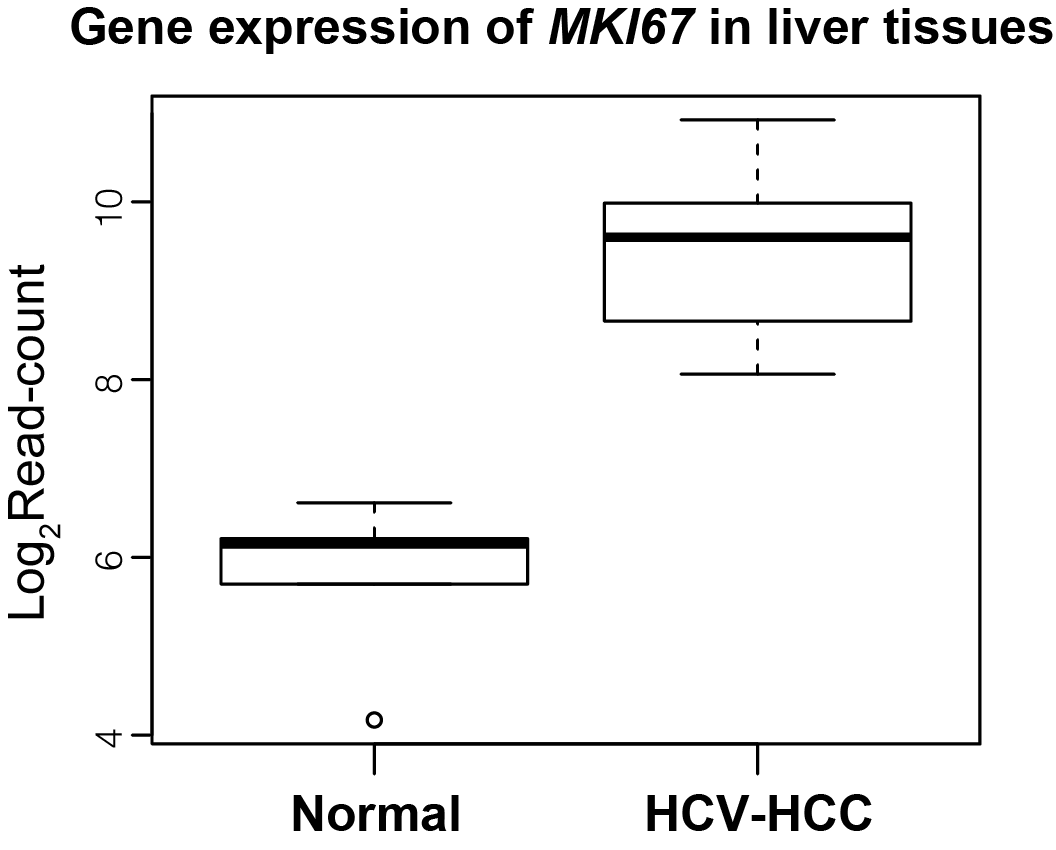

Supplement: Supplementary file 1 — Supplementary figures and tables. [file jcav12p0001s1.zip › Supplementary Figure S2.png]

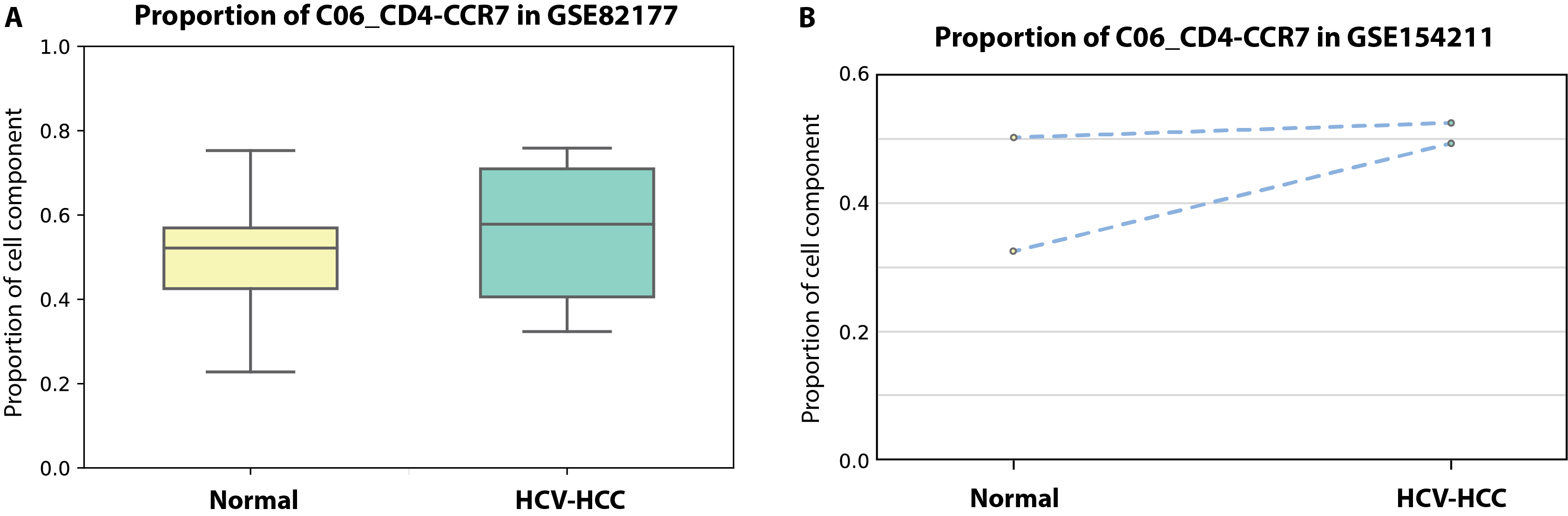

Supplement: Supplementary file 1 — Supplementary figures and tables. [file jcav12p0001s1.zip › Supplementary Figure S3.png]
